# Media coverage of the Ebola virus disease in four widely circulated Nigerian newspapers: lessons from Nigeria

**DOI:** 10.15171/hpp.2016.16

**Published:** 2016-06-11

**Authors:** Sam Smith, Stella Smith

**Affiliations:** ^1^Mass Communication Department, Enugu State University of Science and Technology, Enugu, Nigeria; ^2^Emergency Preparedness and Response Research Group, Nigerian Institute of Medical Research, Lagos, Nigeria

**Keywords:** Ebola, Epidemic, Nigerian newspapers, Media

## Abstract

**Background: ** The importance of the media in the coverage of Ebola virus disease (EVD) in Nigeria and its implications (negative or positive) amongst the populace cannot be overemphasized.This study was conducted to assess the role of media in the Ebola reportage and its implication in creating awareness and stopping the spread amongst the populace.

**Methods:** The nature and extent of media coverage about Ebola in four major national newspapers were examined. The four major national newspapers were The Sun, The Vanguard, The Nation and The Punch newspapers. The period of study ranged from 20 July (when the index case came to Nigeria) to 20 October 2014. Analysis of the newspaper article was according to content.

**Results: ** A total of 1625 articles were published between July 2014 to October 2014 and these were divided into news (1127; 69.4%), features (267; 16.4%), opinion (76; 4.7%), editorials (149; 9.2%) and interviews (6; 0.4%). The most common topic was Ebola cases in Nigeria (17.5%) followed by discrimination due to Ebola (10.8%) and least of all the use of salt and or Kola for the cure of Ebola (5.2%).

**Conclusion: ** Although the World Health Organization (WHO) declared Nigeria Ebola free on the 20th October 2014, continual reportage of the Ebola disease for effective awareness, prevention and control of the virus is recommended.

## Introduction

Although the first reported case of Ebola was in 1976, very little information was passed concerning the outbreak that killed a total of 434 people in Zaire and Sudan.^1^ By 1979 there were three outbreaks of Ebola occurring as a result of two different strains E. Sudan and E. Zaire. Fifteen years after the E. Reston strain was discovered in monkeys but there was no sign of disease in humans.^1^

The most protracted and complicated epidemic of Ebola virus disease (EVD) to date and the first in Western Africa started with 49 cases in Guinea on March 2014 and this majorly and rapidly spread to two other west African countries such as Sierra Leone and Liberia with a total of 28 601 and 11 300 deaths in these three countries alone and accounting for 99.87% of all cases and deaths world-wide as at 30 December 2015.^2^

In Nigeria, the first known case of Ebola came from a Liberian traveler who was already exposed in Liberia while under observation for possible Ebola in Monrovia before coming to Nigeria against medical advice. He arrived Nigeria on the 20th July 2014 acutely ill with symptoms of Ebola but was treated for malaria and when not responsive his blood specimen was taken to LUTH where it was confirmed positive for Ebola and by 25th July he was dead. This triggered a series of events that led to the death of 8 people and 20 cases.

The symptoms of the disease include fever (greater than 38.6°C), severe headache, muscle pain, weakness, diarrhoea, vomiting, abdominal pain, lack of appetite, non-pruritic maculopapular rash. Conjunctival infection and dark red discoloration of the soft palate, hiccups, cough, sore throat, chest pain, difficulty in breathing, difficulty swallowing, progressive worsening of prostration, stupor, hypotension, impaired kidney and liver infection and external haemorrhages.

### Media and Ebola

According to McQuail^3^ when looking at influence of mass media ones focus should rather be on comparing media reality and social reality in certain situations since the diatribe of influences stream from source of media content, consumers of media contents to shape of media contents.^4^ For example according to Sampei and Aoyagi-Usui^5^ increased awareness and concern of climate change amongst the populace was as result of increased media coverage of global warming from January 1998 to July 2007.

A sample of the four major newspapers sampled showed that the stories on Ebola ranged from current information as it pertains to Nigeria with follow up on the occurrences in West Africa as well as outside Africa made the rounds in the stories reported. A new dimension to the Ebola story came from the belief by people that were told to bathe at a certain time with lots of salt as well as drink warm salt so as to prevent Ebola infection also was reported in the newspapers i.e. 8th and 14th August 2014 in Vanguard which started from a rumour spread on the first week of August. This left two people dead and 20 people hospitalized. It is interesting to note that as at the time that two people died from this salt intake, the death rate due to Ebola virus was also two.^6^ The intervention of the government as well as media helped in curbing the spread of this false rumour.

The early reports on Ebola were on closure of schools and late resumption of schools due to the Ebola outbreak nationwide until some necessary measures were put in place to adequately ensure that the outbreak does not spread in schools.

The aim of the study was to assess the role of media in the Ebola reportage and its implication in creating awareness and stopping the spread amongst the populace.

## Materials and Methods

Between 20 July 2014 (when the first index case came to Nigeria) and October 2014 (when the World Health Organization [WHO] declared Nigeria Ebola free), articles about Ebola were identified in *The Punch, The Nation, The Sun* and *The Vanguard* newspapers of Nigeria. These four newspapers are amongst the leading newspapers in Nigeria. Articles that had the word Ebola using some content analysis for ease of analysis was done manually by a single coder with experience in media analysis. In other words, we gave the single coder the different contents to look out for in the Ebola articles and all newspapers were checked physically for these contents as it was difficult to get all the information on the local newspapers on the Internet.

All topics having Ebola or related to Ebola (such as salt intake to help prevent Ebola virus) were also included in the topics. Recorded contents involved news items (whether as breaking or not), editorials, features, opinion and interviews.

Table 1 shows a summary of the newspaper publications on Ebola while the specific content areas are listed in Table 2.

**Table 1:** Summary of newspaper publications of Ebola in Nigeria (n=1625)

**Newspaper**	**News**	**Feature**	**Opinion**	**Editorial**	**Interviews**
*The Sun*	207	58	3	32	2
*The Vanguard*	278	129	30	28	2
*The Nation*	497	64	11	42	1
*The Punch*	145	16	32	47	1
Total	1127 (69.4%)	267 (16.4%)	76 (4.7%)	149 (9.2%)	6 (0.4%)

**Table 2:** Content of the 1625 articles from the four widely read newspapers (July-October 2014)

	n (%)
Cases in Nigeria (Those who have contacted EVD in Nigeria or those being treated)	285 (17.5)
Reported cases of Federal Government Closure of schools due to Ebola	146 (9.0)
Report cases of salt/kolanut intake to prevent EVD (panic, fear, anxiety)	84 (5.2)^a^
Death rates	107 (6.6)
Preparedness (how prepared were Nigerians in combating EVD)	166 (10.2)
Risk of treating patients	168 (10.3)
Funding/cost of fighting Ebola	86 (5.3)
Precautions the public can take	160 (9.9)
Helpers/volunteers	95 (5.9)
Discrimination(those facing discrimination because of having EVD)	176 (10.8)
Ethics (measures to reduce EVD transmission e.g. quarantine)	152 (9.4)

Abbreviations: EVD, Ebola virus disease. ^a^Two deaths were reported from using salt to prevent EVD.

## Results

A total of 1625 articles were published between July 2014 and October 2014 on Ebola outbreak until WHO declared Nigeria Ebola free on the 20th of October 2014. News reportage was the most common (69.4%), followed by features (16.4%), then editorials (9.2%), opinion (4.7%) and least of all interviews (0.4%) (Table 1). The content analysis showed that cases in Nigeria (for those who have contacted Ebola or those being treated in Nigeria) reportage was (17.5%), followed by discrimination against those having EVD (10.8%). The least reported case was the salt/kolanut intake to prevent EVD (5.2%) (Table 2). Other areas that were equally reported were risk of treating patients (10.2%) and preparedness (10.2%). Precautions the public can take (9.9%) as well as measures to reduce transmission in terms of quarantine also featured amongst the reports (9.4%). Federal Government closure of schools due to EVD was also prominent amongst the report (9%) (Table 2).

## Discussion

The media plays a critical role in information dissemination particularly in cases of infectious disease agents occurring at epidemic proportions.^7,8^ The Nigerian media has been lauded in its media coverage of EVD for playing a role in checking its spread by informing the public.^9^ Although the same study suggested that the media should step up their role in interpretative and investigative reportage of disease outbreaks. In addition the studies by Nwanne^7^ and Belo-Osagie^9^ have also commended the Federal Government for taking pro-active measures against the EVD.

From our study, generally the newspapers published mainly news stories followed by feature articles. In terms of content reporting, the reportage from newspapers on those who had contacted Ebola was the highest (17.5%), while intervention by the Federal Government in the closure of schools due to EVD so as to prevent further spread of EVD amongst children was also reported; the least reported content was the issue of funding. This is mainly because most of the government reports were on strategies to improve capacity for planning, prevention and response to EBV or similar crises such as those that came up after EVD in Nigeria e.g. Lassa fever. The funding that was reported were mainly those for combating the EVD scourge in Nigeria as well as those for conducting clinical trials with the aim of finding treatment against EVD and the latter study was to be conducted outside Nigeria since the EVD was quickly contained in Nigeria while other countries such as Liberia, Guinea and Sierra Leone were facing the epidemic in proportions then.

A study by Basch et al^10^ on Ebola coverage of the EVD in three widely circulated newspapers in the United States showed that large emphasis was placed on death tolls and cases in the United States while admitting also that more should be done to educate readers on the epidemic and its transmission.

Of interest was the fact that some people in both rural and urban areas resulted to using salt in combating EVD and this shows the extent information on EVD had reached rural areas in Nigeria and also the fact that even some of the reports carried the fact that the elderly people from the rural areas called on their children to inform them of the ‘salt bath’ as a means to prevent EVD. This is also part of the confusion and anxiety that resulted from EVD reporting. This on its own resulted in two deaths and 5.2% of the reports on EVD carried this trend. This report was eventually found out to be a tasteless joke which spread.^6^

Those who were willing to volunteer to help fight EVD both in Nigeria and outside were also reported but in a smaller proportion (5.9%).

A report by Belo-Osagie^9^ indicted the media in that prior to the EVD in Nigeria, 0.1% of the Nigerian newspapers were devoted to the reportage of the disease and adjudged that it might have been the reason for haphazard response of approach the government and entire populace reacted to it.

A study by Obukoadara and Abuah^11^ on the role and efficiency of the media in performing her surveillance function within the context of EVD came under scrutiny. The authors concluded that the respondents agreed that the media performed her surveillance function in helping to mitigate the EVD scourge but noted however that the radio and TV messages were stronger compared to social, print media etc.

In the month of October, the number of newspaper publications on Ebola reduced drastically, this was probably because there were no more new cases of Ebola outbreak in Nigeria. The month of August recorded the highest number of EVD reportage than other months, while Belo-Osagie^9^ corroborated this report when 50% of the 6-month reportage on EVD was in August alone with two percent reportage occurring in July the month the index case was reported.

From October 2014 when Nigeria was declared Ebola free till January 2016, there has been a total of 32 news reportage on Ebola (mainly November to December 2014) amongst the newspapers studied, with two occurring in January 2016 from *The Vanguard*, while *The Sun* newspaper reported two in January 2016 with one out of the two being an editorial. The current report of January 2016 was on the declaration of the world as Ebola free which was declared by WHO on 14th January and reported 14th and 15th respectively in *The Vanguard* and *The Sun* newspapers.

In a previous report by Belo-Osagie,^9^ 11% of the newspapers under study were on front and back pages and attributed the low prominence to the fact that other issues were contending for more attention such as insecurity and politics. Our study however did not consider this but can corroborate this view of Belo-Osagie^9^ with the low editorials recorded during the Ebola crisis in Nigeria (9.2%).

Phua,^12^ attributed the recent outbreaks of EVD in West Africa particularly, Guinea, Sierra Leone and Liberia to be due to widespread ignorance by lay persons on its cause, transmission and protective measures. The study also further goes to state that consumption of ‘bush meat’, lack of trust for health authorities and its inadequacy as well as traditional funeral practise and inter-border trading may have facilitated the spread.

Although our study compared to the study by Belo-Osagie^9^ looked at greater number of articles it is limited by the limited time frame reviewed as well as the fact that four newspapers were reviewed just like those of Belo-Osagie.^9^ Further limitation in this study could be due to the fact that only a single coder with experience in media analysis completed the content analysis and so the possibility of human error could arise as one has to check each content manually from the newspapers and for each day the article on Ebola appears.

In conclusion, the media played a great role in curbing the spread of the EVD but the media need to do continuous and investigative reportage of disease outbreaks so as to help inform, educate and prepare the populate about infectious diseases and emerging urgent public health issues such as those of Lassa fever that plagued the country much of last year into early this year. There should be more interviews of people knowledgeable in the area of infectious diseases so as to better educate the general public about infectious diseases and emerging urgent health issues.

## Ethical approval

The study was deemed not human subjects research as only articles in the newspapers were analyzed and so approval was not required by the Institutional Review Board.

## Competing interests

The authors declare that there is no conflict of interests.

## Authors contributions

S Sam contributed the newspaper articles that were used in the analysis of the work while S Sam and S Stella drafted and approved the final draft of the article.
